# Anaplastic Carcinoma Arising From a Mural Nodule in Benign Mucinous Cystadenoma

**DOI:** 10.7759/cureus.53603

**Published:** 2024-02-05

**Authors:** Natalia M Barron-Cervantes, Alejandro Martinez-Esteban, Fabiola M Nuccio-Giordano, Regina Faes-Petersen, Alejandro D. G. Gidi, Eduardo Villegas-Tovar

**Affiliations:** 1 Medical School, Universidad Panamericana, Mexico City, MEX; 2 General and Gastrointestinal Surgery Service, Fundación Clínica Médica Sur, Mexico City, MEX; 3 Anesthesia, Fundación Clínica Médica Sur, Mexico City, MEX; 4 General Surgery, Fundación Clínica Médica Sur, Mexico City, MEX; 5 General and Gastrointestinal Surgery, Angeles Health System, Mexico City, MEX

**Keywords:** mesenteric cyst, ascites, pleural effusion, metastasis, mucinous cystadenoma, anaplastic carcinoma

## Abstract

Giant abdominopelvic tumors continue to present a diagnostic and therapeutic challenge for all surgeons despite all the advances in the world of imaging. Particularly, one of the most important challenges is to determine its probable origin for adequate surgical planning. Even though mostly all of these tumors are benign ovarian tumors, extraordinarily, malignant mural nodules may develop from the wall of these benign tumors, carrying an invariable unfavorable prognosis for the patient. This case highlights the importance of a correct diagnostic approach using ultrasound and abdominal computed tomography scans and confirming the diagnosis through a histopathologic examination. The treatment for these cases is surgical resection and posterior oncological treatment if needed. This case shows how timely treatment is one of the principal determinators of morbidity and mortality.

## Introduction

Giant ovarian tumors are uncommon in everyday practice. Most often, the diagnosis is omitted as physicians assume an increase in the abdominal circumference is attributed to two causes, namely, pregnancy and obesity. Due to the advancements in imaging techniques, the incidence of cystic ovarian tumors has increased; however, this advancement also allows early diagnosis, decreasing the incidence of giant ovarian cysts. Nevertheless, they remain the most common cause of giant abdominal tumors in women [1]. The great majority of these cases are mucinous cystadenomas (MCAs). Occasionally, borderline or malignant epithelial tumors are reported. With only a few cases reported in the literature, mucinous neoplasms associated with intramural nodes with different histopathology are even rarer, which makes their diagnosis more difficult, leading to a worse outcome for the patient [2]. Imaging studies in these cases are crucial as they can help formulate a correct presurgical approach that allows the surgeon to be better prepared, with computed tomography (CT) being one of the most used studies due to its high sensitivity. When imaging studies are not sufficient, the importance of histopathological studies and the correct diagnostic approach through the use of stains and immunohistochemistry (IHC) stands out. We present the case of a 68-year-old female patient who presented with a progressive increase in abdominal circumference in a first-level private surgical center in Mexico City. This case is presented to further expand the knowledge about ovarian anaplastic carcinoma presentation within an intramural node of an MCA, as well as highlighting the importance of an early oncological diagnosis that will allow for timely treatment to begin.

## Case presentation

A 68-year-old Latin American woman presented to the gastrointestinal outpatient with a progressive increase in abdominal circumference for three years. She had undergone a total abdominal hysterectomy with bilateral tubal ligation in 2009 and was diagnosed with type 2 diabetes mellitus in 1996, with treatment on regular insulin 20 IU in the morning and 10 U at night and pioglitazone 15 mg PO BID, currently well-controlled with fasting glucose and glycosylated hemoglobin levels within the normal range. She was also diagnosed with systemic arterial hypertension on treatment with losartan 50 mg PO BID. Additionally, no current or past complications were mentioned in her medical history.

**Figure 1:**
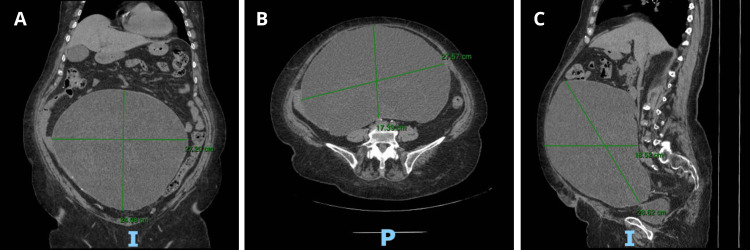
Preoperative abdominal CT scan. The CT scan shows a 27.2 x 24.08 x 18.5 cm abdominal cyst with a probable mesenteric origin with almost complete occupation of the abdominal cavity. (A) Coronal view. (B) Axial view. (C) Sagittal view.

Months earlier, she began to feel a mass in the suprapubic region with progressive growth, for which she decided to visit a doctor at an external clinic where physical exploration revealed the presence of a well-defined tumor in the abdomen. An abdominal CT scan showed a 27.2 x 24.08 x 18.5 cm abdominal cyst with a probable mesenteric origin with almost complete occupation of the abdominal cavity; ovaries were not visible at the time of the study (Figure 1).

During her first consultation at our clinic, the patient mentioned that throughout the year the abdominal circumference had continued to grow to the point where she started to experience dyspnea, orthopnea, and bilateral pitting edema in the lower limbs. During the physical examination, a well-defined suprapubic tumor, mild abdominal pain 4/10 localized on the lower quadrants, peristaltic sounds decreased in frequency and intensity, and 3+ bilateral pitting edema in the lower limbs were noted. During the consultation, she presented her latest laboratory results (Table 1) that showed mild anemia (hemoglobin 10.6 g/dL), leukopenia (2.84 x 10^3^/µL), normal alpha-fetoprotein (2.08 ng/mL), normal cancer antigen 125 (CA-125) (20.74 U/mL), normal carcinoembryonic antigen (CEA) (0.9 ng/mL), and slightly elevated β-subunit human chorionic gonadotropin (3.86 mUI/mL).

**Table 1:** Laboratory results.

Parameter	Value	Reference values
Hemoglobin	10.6 g/dL	12–18 g/dL
Hematocrit	32.5%	36–48%
Platelets	353 × 10^3^/µL	150–450 × 10^3^/µL
Leukocyte count	2.84 × 10^3^/µL	4.5–11 × 10^3^/µL
Absolute neutrophils	1.46 × 10^3^/µL	2.5–7 × 10^3^/µL
Absolute lymphocytes	0.97 × 10^3^/µL	1–4 × 10^3^/µL
Serum glucose	79 mg/dL	70–100 mg/dL
Blood urea nitrogen	15.6 mg/dL	7–20 mg/dL
Urea	33.3 mg/dL	5–20 mg/dL
Serum creatinine	0.61 mg/dL	0.6–1.1 mg/dL
Prothrombin time	9.9 seconds	10–13 seconds
Partial thromboplastin time	28.2 seconds	25–35 seconds
International normalized ratio	0.95 seconds	<1.1 seconds
Alpha fetoprotein	2.08 ng/mL	0–7.0 ng/mL
Cancer antigen 125	20.74 U/mL	0.1–35.0 U/mL
Carcinoembryonic antigen	0.9 ng/mL	0–3.8 ng/mL
β-subunit human chorionic gonadotropin	3.86 mUI/mL	0.0 in men and non-pregnant women

**Figure 2:**
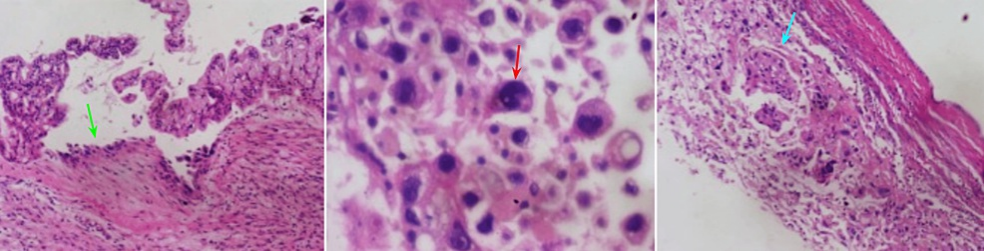
Histopathological findings. Multicystic lesion covered by tall cylindrical cells with endocervical and intestinal-type epithelium (green arrow) with some foci with cylindrical epithelial cells with cellular atypia, large irregular and hyperchromatic nuclei (red arrow), and focal mitoses. Focal nodules of neoplastic cells with intense atypia are identified, with scant cytoplasm and large, irregular, and pleomorphic nuclei; these nodular areas did not infiltrate the ovarian wall or stroma (blue arrow). The findings were indicative of a borderline seromucinous tumor (proliferative atypical mucinous tumor) with intraepithelial carcinoma.

New studies were asked for her follow-up appointment, including CA-125, CEA, and a with-contrast abdominal CT. Before the patient could attend her laboratory and radiology appointment, she was admitted to the emergency room (ER). A week later, she presented to the ER with acute onset dyspnea, low oxygen saturation (82% without oxygen), normal body temperature, tachycardia (130 beats/minute), tachypnea (33 beats/minute), and generalized acute abdominal pain 8/10. An abdominal CT scan was performed where bilateral pleural effusion and ascites were reported. A paracentesis was performed, and 5 L of serosanguineous fluid was obtained. Due to the hemodynamic instability presented by the patient, an urgent laparotomy was performed. During this, a midline incision was made with resection of a giant cystic tumor of the abdomen. During the surgery, it was confirmed that the origin of the tumor was the right ovary, which was not previously noted in any imaging studies. Tumor tissue was sent for a transoperative histopathological study where a borderline seromucinous tumor (proliferative atypical mucinous tumor) with intraepithelial carcinoma was initially reported (Figure 2).

**Figure 3:**
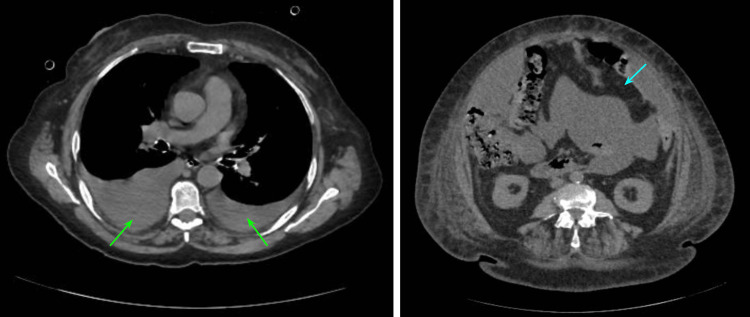
Postoperative abdominal CT scan. The CT scan shows bilateral pleural effusions (green arrows) and abundant ascites fluid (blue arrows).

After the surgery, the patient had an adequate postoperative course and was discharged without complications. A new abdominal CT scan was performed, which showed postoperative changes associated with the previously mentioned surgery, bilateral pleural effusion, and abundant ascites fluid were reported (Figure 3).

**Figure 4:**
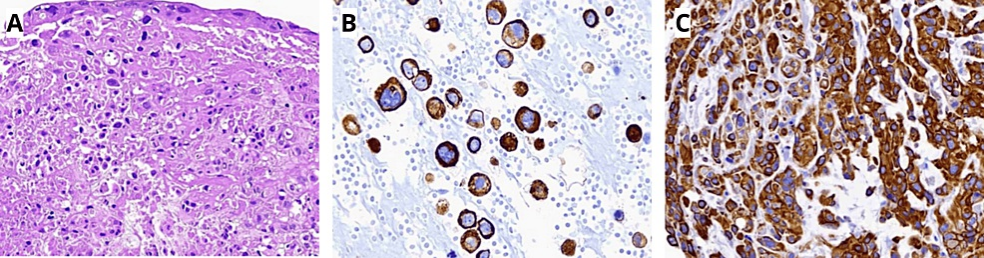
Histopathological findings. Immunohistochemistry study with positive results for CK 5/6 and MUC6. The final diagnosis of anaplastic carcinoma, originating from a mural nodule of borderline mucinous cystadenoma of the right ovary was made. (A) Hematoxylin and eosin stain. (B) Immunohistochemistry positive for CK 5/6. (C) Immunohistochemistry positive for MUC6.

For the definitive histopathological report, the paraffin block corresponding to the right ovarian tumor was further subjected to IHC testing. The test showed a positive result for cytokeratin 5/6 (CK 5/6) and mucin 6 (MUC6). Using data from the first histopathologic study, the IHC testing, tumor size, tumor origin, clinical presentation, and laboratory results, the final diagnosis of an anaplastic carcinoma originating from a mural nodule of borderline MCA of the right ovary was made (Figure 4).

Two months later, the patient presented again to the ER with the same clinical picture as previously presented. A new paracentesis was performed, and this time, it was decided to place a palliative long-term peritoneal catheter drain while oncological treatment began. She was hospitalized to treat any hydroelectrolytic disorder that may present secondary to her state. During her hospitalization, she was stable but generally dehydrated despite intense intravenous hydration therapy secondary to a large amount of fluid lost through drainage secondary to ascites. Her cancer treatment with chemotherapy began later in her hospitalization. However, she died a week later due to acute kidney failure.

## Discussion

Although 7% of women globally have been diagnosed with an ovarian cyst, giant ovarian cysts, which are described in the literature as measuring more than 10 cm in diameter, are an uncommon clinical presentation [3]. These patients not only have a poor prognosis associated with a late diagnostic approach but, in exceptional cases, it has also been associated with malignant tumors that arise from the wall of the benign tumor. This delay has been associated with a low diagnostic suspicion secondary to asymptomatic patients until the tumor is at a more advanced clinical stage. The most common type of tumor associated with giant ovarian cysts is MCA, a benign epithelial tumor. Only in 2018, approximately 22,240 new cases of ovarian cancer were reported, and in the same year, 14,070 deaths secondary to this were reported. Impressively, ovarian cancer accounts for 2.5% of all malignancies among women, and 5% of these are associated with poor survival rates largely driven by late-stage diagnoses [4]. Ovarian epithelial neoplasms represent 60% of all ovarian tumors and 40% of benign tumors. They include a diverse group of neoplasms, with prognoses varying widely depending on their histological characteristics and the stage at which they are detected. Epithelial tumors are classified as benign, representing 80% of all cases, borderline, or malignant tumors. In general, these are more common among women of all racial/ethnic groups, accounting for 90% of all cases. Moreover, these tumors may be classified according to the histology of the tumor cells as serous (52%), endometrioid (10%), mucinous (6%), or clear cell (6%), with a quarter classified as rarer subtypes or not specified [5].

It has been proven that mucinous tumors progress naturally from benign to borderline and eventually to malignant. The hypothesis is that this is due to *KRAS* mutations, which lead to a specific biological behavior that allows the cells to continue to mutate even after forming the initial tumor [6]. MCA is considered a benign, non-invasive tumor, which can present atypia without stromal involvement. It represents 80% of mucinous tumors and usually occurs in women between the third and sixth decade of life, and in 90% of all cases it occurs unilaterally. Macroscopically, they have a smooth surface and are multilocular, with sizes varying widely from a few centimeters to 30 cm with an average of 10 cm. Microscopically, they are multicystic tumors with abundant mucin inside and little extracellular mucin, unlike tumors of gastrointestinal origin with metastasis to the ovary, which present abundant extracellular mucin [7]. When describing the histopathological findings, many patterns have been identified, presenting the main characteristic of an inherent glandular complexity of proliferating mucinous cells. Invasion is usually very difficult to discriminate from carcinomas. However, in the literature, mural nodules found in the cystic wall tend to be a very rare finding and have a reactive or neoplastic pathology [8]. They can be classified into the following five histological patterns: leiomyoma, sarcomas, sarcoma-like, anaplastic carcinoma, and hybrid features. It is very important to mention that anaplastic carcinoma is almost always associated with MCA or borderline tumors. This histological pattern is associated with metastasis in the lung and myocardium. Its histopathological features remain a diagnostic challenge [4]. The most common tumor marker associated with ovarian cancer is CA-125 followed by CA-199. However, many cases of anaplastic carcinoma present CEA, CA-125, and CA-199 within normal limits, which makes it more difficult to suspect and eventually diagnose [9]. This correlates with the present case.

As previously mentioned, the correct diagnostic approach to giant abdominal tumors is of vital importance. The use of abdominal CT scans has been standardized over the years associated with its high sensitivity; however, its low specificity for evaluating pelvic structures presents a diagnostic challenge. The use of abdominal ultrasound (US) is preferred for both screening and addressing ovarian tumors. However, the presence of an abdominal giant tumor, as in this case, makes the use of diagnostic US almost impossible due to the size of the tumor relative to the capacity of the US to show the tumor in its complete extension [10]. Nevertheless, it is important to mention that the use of abdominal CT is vital in these cases for a preoperative evaluation that will allow the surgeon to formulate a surgical plan. In every surgical case, it is important to perform a transoperative and definitive histopathological study; however, in ovarian tumors, it is the most relevant part of the diagnostic approach. Not only transoperative but definitive histopathological study with IHC is vital in diagnosing such tumors. MUC6 is a mucin studied in gastric carcinomas; however, mucins are synthesized by any secretory epithelial cell, explaining why an ovarian epithelial carcinoma expresses it. The expression of MUC6 and MUC2 has been associated with the progression of benign to malignant tumors, which has been attributed to the malignant change [11]. On the other hand, the expression of CK 5/6 has been proven to be used as a marker for carcinomas and its expression is useful in separating benign from malignant epithelial tumors [12]. Other IHC markers that have been used in literature are cytokeratin, epithelial membrane antigen, vimentin, and integrase interactor 1 [13].

## Conclusions

Abdominal giant tumors have a high probability of presenting an ovarian origin. A timely diagnosis of ovarian tumors not only reduces morbidity but also mortality associated with cancer detected in late stages. The use of the US has been limited to ovarian tumors larger than 10 cm. The low specificity presented by the CT scan can be associated with late diagnoses. However, its high sensitivity serves greatly as a preoperative study, as it allows proper surgical planning. It is crucial to emphasize that the diagnostic approach through imaging studies must always be complemented by a complete histopathological study, including IHC. The expression of molecules secreted by epithelial cells has not only facilitated pathological diagnosis but is now considered an indicator of malignancy. As mentioned, these molecules have been associated with the progression of benign to malignant tumors, helping as a diagnostic tool in cases where the biopsy may present a complex pattern difficult to diagnose, or cases like this, where two histopathological diagnoses can be found within the same tumor. Even though the presentation of malignant tumors within the wall of giant benign cystic tumors has been presented as anecdotal in literature, it must always be ruled out in cases where the tumor has been evolving for a long time, similar to this case. The fatal outcome of this case proves that a timely diagnosis leads to an early oncological approach, the most important factor in patient survival.
